# Expert consensus on the diagnosis and treatment of macrolide-resistant *Mycoplasma pneumoniae* pneumonia in children

**DOI:** 10.1007/s12519-024-00831-0

**Published:** 2024-08-14

**Authors:** Ying-Shuo Wang, Yun-Lian Zhou, Guan-Nan Bai, Shu-Xian Li, Dan Xu, Li-Na Chen, Xing Chen, Xiao-Yan Dong, Hong-Min Fu, Zhou Fu, Chuang-Li Hao, Jian-Guo Hong, En-Mei Liu, Han-Min Liu, Xiao-Xia Lu, Zheng-Xiu Luo, Lan-Fang Tang, Man Tian, Yong Yin, Xiao-Bo Zhang, Jian-Hua Zhang, Hai-Lin Zhang, De-Yu Zhao, Shun-Ying Zhao, Guo-Hong Zhu, Ying-Xue Zou, Quan Lu, Yuan-Yuan Zhang, Zhi-Min Chen

**Affiliations:** 1 Department of Pulmonology, Children’s Hospital, Zhejiang University School of Medicine, National Clinical Research Center for Child Health, Hangzhou 310052, China; 2 Department of Child Health Care, Children’s Hospital, Zhejiang University School of Medicine, National Clinical Research Center for Child Health, Hangzhou 310052, China; 3 Department of Pediatric Pulmonology and Immunology, West China Second University Hospital, Sichuan University, Chengdu 610041, China; 4 Department of Pediatrics, Shandong Provincial Hospital Affiliated to Shandong First Medical University, Jinan 250021, China; 5 Department of Pulmonology, Shanghai Children’s Hospital, School of Medicine, Shanghai Jiao Tong University, Shanghai 200062, China; 6 Department of Pulmonary and Critical Care Medicine, Kunming Children’s Hospital, Kunming 650034, China; 7 Department of Respiratory Medicine, Children’s Hospital of Chongqing Medical University, Chongqing 400014, China; 8 Department of Pulmonology, Children’s Hospital of Soochow University, Suzhou 215003, China; 9 Department of Pediatrics, Shanghai General Hospital Affiliated to Shanghai Jiaotong University School of Medicine, Shanghai 200080, China; 10 Department of Respiratory Medicine, Wuhan Children’s Hospital, Tongji Medical College, Huazhong University of Science and Technology, Wuhan 430015, China; 11 Department of Respiratory Medicine, Children’s Hospital of Nanjing Medical University, Nanjing 210008, China; 12 Department of Respiratory Medicine, Shanghai Children’s Medical Center, Shanghai Jiao Tong University School of Medicine, Shanghai 200127, China; 13 Department of Respiratory Medicine, Children’s Hospital of Fudan University, Shanghai 201102, China; 14 Department of Pediatric Pulmonology, Xinhua Hospital, Shanghai Jiaotong University School of Medicine, Shanghai 201102, China; 15 Department of Pediatric Respiratory Medicine, The Second Affiliated Hospital and Yuying Children’s Hospital of Wenzhou Medical University, Wenzhou 325027, China; 16 National Clinical Research Center for Respiratory Diseases, Beijing Children’s Hospital, Capital Medical University, Beijing 100045, China; 17 Department of Pulmonology, Tianjin Children’s Hospital (Children’s Hospital of Tianjin University), Tianjin 300074, China

**Keywords:** Child, Macrolide-resistant *Mycoplasma pneumoniae*, Recommendation, Diagnosis, Treatment

## Abstract

**Background:**

*Mycoplasma pneumoniae* (*M. pneumoniae*) is a significant contributor to community-acquired pneumonia among children. Since 1968, when a strain of *M. pneumoniae* resistant to macrolide antibiotics was initially reported in Japan, macrolide-resistant *M. pneumoniae* (MRMP) has been documented in many countries worldwide, with varying incidence rates. MRMP infections lead to a poor response to macrolide antibiotics, frequently resulting in prolonged fever, extended antibiotic treatment, increased hospitalization, intensive care unit admissions, and a significantly higher proportion of patients receiving glucocorticoids or second-line antibiotics. Since 2000, the global incidence of MRMP has gradually increased, especially in East Asia, which has posed a serious challenge to the treatment of *M. pneumoniae* infections in children and attracted widespread attention from pediatricians. However, there is still no global consensus on the diagnosis and treatment of MRMP in children.

**Methods:**

We organized 29 Chinese experts majoring in pediatric pulmonology and epidemiology to write the world’s first consensus on the diagnosis and treatment of pediatric MRMP pneumonia, based on evidence collection. The evidence searches and reviews were conducted using electronic databases, including PubMed, Embase, Web of Science, CNKI, Medline, and the Cochrane Library. We used variations in terms for “macrolide-resistant”, “*Mycoplasma pneumoniae*”, “MP”, “*M. pneumoniae*”, “pneumonia”, “MRMP”, “lower respiratory tract infection”, “*Mycoplasma pneumoniae* infection”, “children”, and “pediatric”.

**Results:**

Epidemiology, pathogenesis, clinical manifestations, early identification, laboratory examination, principles of antibiotic use, application of glucocorticoids and intravenous immunoglobulin, and precautions for bronchoscopy are highlighted. Early and rapid identification of gene mutations associated with MRMP is now available by polymerase chain reaction and fluorescent probe techniques in respiratory specimens. Although the resistance rate to macrolide remains high, it is fortunate that *M. pneumoniae* still maintains good in vitro sensitivity to second-line antibiotics such as tetracyclines and quinolones, making them an effective treatment option for patients with initial treatment failure caused by macrolide antibiotics.

**Conclusions:**

This consensus, based on international and national scientific evidence, provides scientific guidance for the diagnosis and treatment of MRMP in children. Further studies on tetracycline and quinolone drugs in children are urgently needed to evaluate their effects on the growth and development. Additionally, developing an antibiotic rotation treatment strategy is necessary to reduce the prevalence of MRMP strains.

## Introduction

*Mycoplasma pneumoniae* (*M. pneumoniae*) is an important pathogen of respiratory infections in children, accounting for 20%–40% of pediatric community-acquired pneumonia, with even higher rates in older children and during the epidemic season. Generally, *M. pneumoniae* infections have a certain degree of self-limitation, with most cases being mild with a good prognosis. Macrolide antibiotics are the preferred medication for *M. pneumoniae* infections in children. Although tetracycline and respiratory quinolone antibiotics have similar clinical efficacy against *M. pneumoniae*, their potential side effects limit their application in children, especially young children.

Since 2000, macrolide-resistant *M. pneumoniae* (MRMP) infections have become more prevalent around the world, especially in East Asia, where the isolation rate of MRMP has reached as high as 70%–90% [1]. This high prevalence has posed significant challenges for pediatricians. In the absence of effective antibiotics, MRMP infections inevitably bring a series of clinical problems, including prolonged fever and hospitalization, an increase in severe cases, and difficulties in antibiotic selection. Yet, there still remains a lack of global consensus on the diagnosis and treatment of MRMP in children. Therefore, we organized 29 Chinese experts majoring in pediatric pulmonology and epidemiology to develop the world’s first consensus on the diagnosis and treatment of pediatric MRMP pneumonia, aiming to provide scientific guidance for the clinical practice of pediatricians.

This consensus was formulated by the National Clinical Research Center for Child Health (Children’s Hospital, Zhejiang University School of Medicine). Based on an extensive literature review and three rounds of online Delphi voting, a consensus addressing 11 clinical questions was approved and agreements on recommendations were achieved. These include various aspects of MRMP pneumonia, including epidemiology, disease burden, resistance mechanisms, antibiotic sensitivity, clinical futures, early recognition, laboratory confirmation, principles of antibiotic therapy, application guidelines for flexible bronchoscopy, glucocorticoids, and immunoglobulins.

## Methods

### Design

The consensus process applied a Delphi method that is a reliable and validated technique to reach consensus on topics where information is insufficient or where there is an overload of contradictory information [2, 3]. A three-round series of statement drafts were voted on via a Chinese online application (https://www.wjx.cn/) between January and March 2024 by 28 experts in pediatric pulmonology, recruited from across China.

### Consensus formulation working group

The consensus formulation working group comprises the expert group and the consensus secretariat group. The consensus expert group consisted of 28 pediatric pulmonologists from major hospitals nationwide, primarily responsible for selecting clinical issues, revising consensus opinions, and achieving consensus through three rounds of voting panels. Fifteen (55.6%) of them were female; the age ranged from 36.8 to 82.0 years, with an average age of 54.6 years and a standard deviation (SD) of 9.1. Twenty-one (77.8%) held a doctorate degree, four (14.8%) a master’s degree, and two (7.4%) a bachelor’s degree. The time of expertise as a pediatrician majoring in pulmonology ranged from 9 to 51 years, with an average time of 31.2 years and a SD of 10.3.

The consensus secretariat group consisted of seven members, including three senior pediatricians who specialized in pediatric pulmonology with doctoral degrees, an epidemiologist with a doctoral degree who had expertise in methodology of evidence-based medicine, and three master’s students who also specialized in pediatric pulmonology. The main responsibilities of the core group were as follows: (1) conducting literature reviews; (2) identifying main clinical topics; (3) elaborating statements on each topic; (4) coordinating three rounds of online consensus panels; (5) data collection and analyzing; and (6) documenting the expert consensus formulation process.

### Process of reaching consensus

Before the first round of Delphi voting, the consensus secretariat group proposed four main clinical topics (epidemiology, diagnosis, treatment, and prevention of MRMP in children) with 11 clinical questions based on literature reviews and experts’ opinions. In the first round, the expert panel members were invited to rate the four main topics and 11 questions regarding their importance and operability using a numerical Likert scale with five options. The expert panel participants were encouraged to give comments on each question and propose new questions. The clinical question was retained when the mean score of importance and operability was higher than 3.5 and the coefficient of variation was less than 0.30. The question(s) were modified, added, or deleted according to the experts’ comments. Consequently, the experts in the secretariat group added one main topic (i.e., pathogenic characteristics and pathogenesis of MRMP in children) and two clinical questions to the second-round questionnaire. After the two rounds of Delphi voting, consensus was reached by excluding one main topic and two questions. Thus, four topics and 11 questions were retained for the last round of voting. Experts in the secretariat group proposed recommendation opinions for each clinical question for the last round of online voting by the expert panel members. There were three options: agree, disagree, and not sure. The agreement on recommendation was made when more than two-thirds of the expert panel members chose “agree”.

## Results

### Epidemiology

#### Global trend of MRMP

##### Recommendations

The prevalence of MRMP varies significantly across different regions globally. Since 2000, the proportion of MRMP has been gradually increasing in the Western Pacific region, especially in China, South Korea, and Japan, while Japan experienced a decline in MRMP after 2012. In other regions of the world, MRMP prevalence has remained at a low level.

##### Summary of the evidence

Surveillance in Beijing at different time periods revealed a high macrolide resistance rate of 90.6% (280/309) during 2008–2012 [4]. From 2010 to 2012, it was 88.3% (181/205), with a higher rate of 94.3% (83/88) observed in children] [5]. During 2016–2019, the resistance rate was 90.94% (1386/1524) [6]. In Shanghai, from 2005 to 2008, 83% (44/53) of isolated strains showed macrolide resistance, which increased to 90% (90/100) from 2008 to 2009 [7]. During 2021–2022 in Beijing, the MRMP rate in hospitalized children reached as high as 92.7% (482/520) [8].

Morozumi et al. [9] found that in Japan, from 2002 to 2005, the macrolide resistance rate of *M. pneumoniae* was 6.9% (18/259), increasing to 37.4% (96/257) from 2006 to 2009, 86.2% (281/326) from 2010 to 2013, 56.3% (111/197) in 2015–2016, and 11.3% (6/53) in 2018–2019. Another study found that from 2008 to 2015, the macrolide resistance rate of *M. pneumoniae* in Japanese children ranged from 43.6% in 2015 to 81.6% in 2012, with the highest rate observed in 2012 [10]. The overall prevalence of MRMP in Japan from 2008 to 2018 was 68.6% [11].

In Taiwan Province of China, before 2017, the average resistance of macrolides was about 15%–30%, but since 2018, the prevalence of MRMP has rapidly increased from 54.3% (44/81) between 2016 and 2019 to 77% (174/226) between 2017 and 2019 [12, 13]. In Korea, MRMP has shown a significant increasing trend, from 4% before 2008 to 78% between 2015 and 2017 [14]. From 2019 to 2020, the proportion of MRMP pneumonia in Korea continued to rise, with an overall macrolide resistance rate of 78.5% (73/93) [15].

In Europe, North America, South America, and Oceania, the overall prevalence rates of MRMP are 3%, 8.6%, 0%, and 3.3%, respectively. Yamada et al. [16] found an MRMP incidence rate of 8.2% (4/49) among children in St. Louis, Missouri, USA, between 2007 and 2010. From 2012 to 2014, the MRMP incidence rate in six regions of the United States was 13.2% (12/91) [17]. Between 2015 and 2018, the MRMP incidence rate in eight states of the United States was 7.5% (27/360) [18]. From 2014 to 2021, the macrolide resistance rate of *M. pneumoniae* infections in children and adults in the Midwestern United States was 9.6% (11/114) [19]. During the outbreak of *M. pneumoniae* infection in Italy in 2010, the MRMP incidence rate among hospitalized children was 26% (11/43) [20]. A retrospective analysis of *M. pneumoniae* infections in children and adults in Spain from 2013 to 2017 showed an MRMP incidence rate of 8% [21]. In Scotland, the MRMP incidence rate was 19% in 2010–2011, while in England and Wales, it was 9.3% in 2014–2015. In France, the incidence rate of MRMP was 3.4%–8.3% between 2007 and 2010, and in Germany, it was 3.65% between 2009 and 2012, and 3% between 2016 and 2018. MRMP incidence rates in Slovenia, Sweden, and Denmark were all < 3%, while no MRMP was detected in Finland and the Netherlands [22].

#### Disease burden of childhood MRMP pneumonia

##### Recommendations

MRMP pneumonia significantly increases the disease burden and healthcare costs.

##### Summary of the evidence

In pediatric respiratory tract infections, *M. pneumoniae* is a significant pathogen, accounting for 40% of total cases of community-acquired pneumonia in children and 19% of cases requiring hospitalization [23]. MRMP can complicate treatment, and ineffective antimicrobial therapy may lead to pneumonia progression or more extrapulmonary complications [24]. Among patients with MRMP infection, prolonged fever duration, extended hospital stays, heightened oxygen requirements, elevated need for intensive care unit admission, prolonged courses of antimicrobial therapy, and a higher likelihood of requiring alternative antimicrobial agents have been observed [23, 25–27]. The likelihood of admission to the pediatric intensive care unit is also increased by fivefold in patients with MRMP infection [23]. MRMP pneumonia often exhibits clustering or outbreak patterns [28–31] leading to transmission within schools, communities, among close contacts, and within households, imposing significant socioeconomic burdens.

### Mechanisms

#### Resistance mechanisms of MRMP

##### Recommendations

MRMP is associated with point mutations in domain V of the 23S rRNA gene of *M. pneumoniae*, particularly mutations corresponding to the A2063G or A2064G transitions. The emergence of MRMP has close association with the widespread use of macrolide antibiotics.

##### Summary of the evidence

In 1968, Niitu et al. [32] from Japan first isolated highly resistant *M. pneumoniae* from a girl with pneumonia treated with erythromycin. In 2001, Okazaki et al. [33] from Japan reported the nucleotide mutations associated with macrolide resistance in *M. pneumoniae*. It is now recognized that mutations in the ribosomal target genes of antimicrobial agents are definitive in MRMP, and acquired mutations leading to modifications in antimicrobial target sites are associated with antimicrobial resistance [34]. A global meta-analysis of MRMP found that the most common mutations in MRMP pneumonia are A2063G, followed by A2064G [1]. Although the proportion of MRMP pneumonia associated with the A2064G variant is relatively low, countries such as Cuba, Germany, Italy, and Switzerland show higher proportions of MRMP pneumonia associated with this variant (> 30%) [1]. Other rare mutations associated with MRMP pneumonia include A2063C, A2063T, A2064C, C2617A, C2617G, A2067G, A2054G, A2058G, and A1290G [1, 35]. Point mutations in domain II of the 23S rRNA gene, the* rplD* gene (encoding ribosomal protein L4), and the *rplV* gene (encoding ribosomal protein L22) may also be associated with macrolide resistance [36]. The active efflux mechanism may also contribute to the development of macrolide resistance in *M. pneumoniae* [37].

*M. pneumoniae* isolates carrying the A2063G and A2064G mutations exhibit significantly high minimum inhibitory concentrations (MICs) for 14- and 15-membered macrolides, with MICs for 14-membered macrolides reaching 256 mg/L, and for 15-membered macrolides, ranging from 16 to 64 mg/L. However, these isolates have lower MICs for 16-membered macrolides, ranging from 0.0156 to 16 mg/L [38]. Other point mutations in *M. pneumoniae* isolates, such as C2617A or C2617T, have lower MICs for macrolides compared to those with A2063G and A2064G mutations (0.0313–8 mg/L) [34].

The overuse of macrolide antibiotics is believed to be associated with the emergence of MRMP [39]. Studies have shown a correlation between the widespread use of macrolides in the Western Pacific region and the high prevalence of MRMP [40]. There are also reports in the literature that some isolates that were originally sensitive to macrolides later became MRMP [20].

#### Sensitivity of MRMP to antibiotics

##### Recommendations

*M. pneumoniae* strains with MIC values < 0.5 mg/L for erythromycin and azithromycin are considered sensitive, whereas those with MIC values > 1 mg/L are considered resistant. Currently, most clinical isolates of MRMP have MIC values ≥ 128 and ≥ 2 mg/L for erythromycin and azithromycin, respectively. *M. pneumoniae* strains are considered sensitive for tetracycline and doxycycline when MIC values are ≤ 2 mg/L, and for minocycline ≤ 4 mg/L. Currently, MRMP isolates are sensitive to both doxycycline and minocycline. Among quinolone antibiotics, levofloxacin is considered sensitive when the MIC value is ≤ 1 mg/L, and for moxifloxacin ≤ 0.5 mg/L. Currently, MRMP isolates are all sensitive to quinolone antibiotics, such as levofloxacin, moxifloxacin, and tosufloxacin.

##### Summary of the evidence

In a study conducted in Beijing [41], out of 81 strains of isolated *M. pneumoniae* from 2014 to 2016, 53 (65.4%) showed resistance to erythromycin (MIC: ≥ 256 mg/L) and azithromycin (MIC: 2–64 mg/L) in vitro. The remaining 28 strains were sensitive to macrolides, with MIC values ≤ 0.008 mg/L for both erythromycin and azithromycin. All 81 clinical isolates of *M. pneumoniae* were sensitive to tetracycline (MIC: 0.016–0.5 mg/L) and levofloxacin (MIC: 0.125–1 mg/L).

In a multicenter study conducted in five cities of China in 2018 (Jilin, Beijing, Jinan, Fuyang, and Suzhou) [42], 79.9% (123/154) of the isolated *M. pneumoniae* strains showed resistance to macrolides, with erythromycin MIC ranging from 128 to > 256 mg/L and azithromycin MIC ranging from 2 to 32 mg/L. The highest macrolide resistance rate was observed in Jilin (100%), followed by Suzhou (91.7%), Fuyang (75%), Beijing (66.7%), and Jinan (54.5%). This study also highlighted significant regional differences in macrolide resistance among *M. pneumoniae* strains. The remaining 31 *M. pneumoniae* strains were sensitive to macrolides, with MIC values ≤ 0.008 mg/L for both erythromycin and azithromycin. All isolates were sensitive to tetracycline (MIC: 0.016–0.5 mg/L) and levofloxacin (MIC: 0.125–1 mg/L).

Another study isolated 182 strains of *M. pneumoniae* from 2017 to 2019 in Shanghai [43]. Of these, 177 (97.3%) strains were resistant to erythromycin (MIC: ≥ 64 mg/L), while only 5 (2.7%) strains were sensitive to macrolides, with MIC values ≤ 0.125 mg/L. All 182 clinical isolates of *M. pneumoniae* were sensitive to tetracycline (MIC: 0.06–2 mg/L), doxycycline (MIC: 0.015–1 mg/L), and fluoroquinolones (MIC: ≤ 0.06–1 mg/L). The range of minocycline MIC was 0.03–4 mg/L, and moxifloxacin (MIC: 0.015–0.25 mg/L) showed greater activity compared to levofloxacin (MIC: 0.03–1 mg/L).

In a multicenter study conducted in Japan [44], which collected acute *M. pneumoniae* infection cases from eight regions in Japan from 2011 to 2016, 1256 strains of *M. pneumoniae* were isolated. Among these strains, 873 were resistant to macrolides, with erythromycin MIC ranging from 32 to > 128 mg/L and azithromycin MIC ranging from 0.25 to > 128 mg/L. However, all isolates were sensitive to tetracyclines (MIC: 0.125–1 mg/L) and fluoroquinolones (MIC: 0.25–1 mg/L).

Another study from Japan isolated 122 strains, including 76 macrolide-sensitive *M. pneumoniae* (MSMP) and 46 MRMP strains, and analyzed the MIC of *M. pneumoniae* strains from 2017 to 2020 [45]. MRMP strains showed varying MIC values for different antibiotics, with MIC values for erythromycin ranging from 16 to > 128 mg/L and for azithromycin ranging from 64 to > 128 mg/L. All *M. pneumoniae* isolates were sensitive to minocycline and levofloxacin.

In a multicenter study conducted in the United States, which included *M. pneumoniae*-infected individuals from nine states from 2012 to 2018, 92.7% of 323 isolated *M. pneumoniae* strains showed MIC values of < 0.008 mg/L for erythromycin, with only 7.3% having MIC values of > 8 mg/L [46].

### Diagnosis

#### Clinical features of MRMP pneumonia

##### Recommendations

While most cases of *M. pneumoniae* infection are mild and self-limiting, some patients may progress to pneumonia and severe cases with pulmonary and/or extrapulmonary complications. The clinical efficacy of macrolide treatment in MRMP patients tends to be lower than in MSMP patients. The initial symptoms, laboratory findings, and imaging manifestations of MRMP pneumonia are similar to those of MSMP pneumonia. However, MRMP pneumonia tends to present with a prolonged fever, require a longer duration of antibiotics, higher rates of antibiotic shift, and increased corticosteroid need compared to MSMP pneumonia.

##### Summary of the evidence

Whether MRMP pneumonia follows a different clinical course to MSMP pneumonia is still under debate. In an early study, Suzuki et al. [47] found that MRMP patients experienced longer total and febrile days after macrolide therapy when compared to MSMP patients (median: 8 vs. 5 days, *P* = 0.019; 3 vs. 1 day, *P* = 0.002). Additionally, MRMP-infected patients were more likely to switch from initial macrolide prescriptions to alternative antibiotics [63.6% vs. 3.8%, odds ratio (OR) = 43.8, *P* < 0.001], suggesting poor efficacy of macrolides against MRMP. However, fever resolved even without changing the initial prescription. In subsequent studies, it was mostly reported that MRMP-infected patients exhibited longer fever durations, increased hospitalization rates [48], and higher levels of C-reactive protein (CRP) [49], but without any significant difference in severity or extrapulmonary complications [48–54].

Minority studies revealed the difference in severity and complications between MRMP pneumonia and MSMP pneumonia. Zhou et al. [55] found that MRMP-infected patients experienced more complications, including hepatic dysfunction, myocarditis, rash, encephalitis, proteinuria, hemolytic anemia, and arthritis, in addition to significantly longer median fever durations and delayed defervescence after macrolide treatment compared to MSMP-infected patients. Kim et al. [56] reported similar fever durations and no differences in leukocyte count, CRP, or erythrocyte sedimentation rate (ESR) between the two groups, except for more cases of pleural effusion in the MRMP group. Surveillance data from Japan also show that as the incidence of childhood MRMP pneumonia increases, there has also been a gradual rise in the number of patients requiring hospitalization [57]. However, some other studies reported similar fever durations and no differences in clinical symptoms, laboratory tests such as leukocyte count, CRP, or ESR, and imaging findings between MRMP and MSMP infections [56, 58].

Recently, a systematic review and meta-analysis revealed no difference in the clinical severity between MRMP and MSMP pneumonia, but MRMP-infected patients had longer fever periods (1.71 days), hospital stays (1.61 days), antibiotic treatment durations (2.93 days), and post-macrolide defervescence times (2.04 days) compared to MSMP-infected patients. The risk of fever lasting > 48 hours after macrolide treatment also significantly increased (OR = 21.24), as did the proportion of patients switching to second-line treatment (OR = 4.42) [27].

Based on the above-mentioned evidence, MRMP pneumonia seems to have similar initial symptoms, laboratory findings, and imaging manifestations compared to MSMP pneumonia, and does not increase the severity of the disease or the risk of complications. However, the clinical efficacy of macrolide treatment in MRMP patients tends to be lower than in MSMP patients. Without early and effective anti-*M. pneumoniae* therapy, MRMP-infected children will exhibit prolonged fever and other symptoms, causing extended hospital stays, longer antibiotic usage, and more frequent corticosteroid usage. Furthermore, there is an increased risk of disease progression, severe pneumonia, and pulmonary and/or extrapulmonary complications.

#### Early identification of MRMP pneumonia in clinical practice

##### Recommendations

Children with *M. pneumoniae* pneumonia (MPP) should be monitored closely for the possibility of MRMP if they continue to have fever or worsening condition despite three days of macrolide therapy. Serum lactate dehydrogenase (LDH), D-dimer, and other inflammatory markers might have some early predictive value for MRMP pneumonia; however, it is not reliable to determine *M. pneumoniae* resistance relying solely on individual laboratory markers, clinical symptoms, chest imaging findings, and bronchoscopy findings.

##### Summary of the evidence

In general, macrolide antibiotics are effective for MSMP pneumonia, and fever usually subsides within 48–72 hours. However, in cases of MRMP pneumonia, fever usually persists for more than 48–72 hours despite macrolide administration, implying that children with MPP who continue to have fever after three days of macrolide treatment should be highly vigilant for the possibility of MRMP infection [13, 59]. In the 2023 Chinese guideline for the diagnosis and treatment of MPP in children, the concept of macrolide-unresponsive MPP had been proposed, referring to cases with MPP failing to show clinical or radiological improvement after 72 hours of macrolide treatment [60].

In addition to the clinical efficacy of macrolide, there has been significant research interest in the use of biomarkers to assist in determining MRMP in recent years. By comparing clinical data between MRMP and MSMP, Narita et al. [61] found that elevated LDH may provide some indication for MRMP. Due to the amplification effect of proinflammatory cytokines such as interleukin (IL)-8 and IL-18, MRMP infection might elicit a persistent and intense immune response, with LDH being considered a more sensitive immunological marker than CRP [8, 62]. Matsuda et al. [63] found significantly higher serum levels of interferon-γ (IFN-γ), IL-6, and IFN-γ-induced protein 10 in MRMP patients compared to MSMP-infected individuals. Wu et al. [64] reported no significant differences in clinical symptoms, hospitalization duration, and imaging findings between MRMP and MSMP pneumonia, but MRMP-infected patients had significantly higher levels of IL-13 and IL-33. Logistic regression analysis identified that a higher white blood cell count was more sensitive for identifying MRMP, with the optimal threshold being 8.55 × 10^9^/L, while a higher D-dimer level was more specific, with the optimal threshold being 523 μg/L [65]. These studies indicated that routine blood tests and cytokine levels might serve as potential candidate markers for predicting MRMP infection.

Although fiberoptic bronchoscopy is an invasive procedure, it does play an important role in the treatment of severe and refractory *M. pneumoniae* pneumonia (RMPP), especially in children with severe airway obstruction. Limited research has indicated differences in mucosal manifestations under bronchoscopy between MRMP pneumonia and MSMP pneumonia. It was found that MRMP pneumonia mainly manifested as mucosal erosion, necrosis, bronchial stenosis, and a higher likelihood of endogenous plastic plugs or cast formation. In contrast, MSMP pneumonia was characterized by mucosal longitudinal folds, fluffy secretions, and viscous secretions. These observations suggest that the overall bronchoscopic manifestations in MRMP-infected children are more severe [65, 66]. However, more evidence is needed to support these findings.

#### Laboratory methods for confirming MRMP

##### Recommendations

It is recommended to use polymerase chain reaction (PCR) and fluorescent probe technology for the rapid diagnosis of MRMP by detecting point mutations associated with macrolide resistance in *M. pneumoniae*. In conditional medical units, performing in vitro cultivation of *M. pneumoniae* and drug susceptibility testing is recommended to analyze the resistance of *M. pneumoniae* to antimicrobial drugs.

##### Summary of the evidence

The standard method for diagnosing MRMP involves isolating *M. pneumoniae* through culture from clinical samples and conducting drug sensitivity tests. However, *M. pneumoniae* culture is time consuming, typically taking at least two weeks to complete. Furthermore, when compared to serological tests or molecular techniques including PCR, culture sensitivity may be lower than 60%–70% [67–69]. Therefore, culture methods are rarely used for routine diagnosis of *M. pneumoniae* infection in clinical practice, except in conditional medical units particularly for research purposes.

To optimize clinical decisions in the treatment of MPP, early detection of MRMP is necessary. Molecular diagnostic techniques make it possible because of the advantages in speed, sensitivity, and specificity. Commonly amplified target genes in experiments usually include the P1 protein-encoding gene, 16S rRNA, *CARDS* gene, and *ATPase* gene. Based on these genes, various molecular methods have been developed to rapidly detect MRMP in clinical samples. In addition to traditional PCR and 23S rRNA gene sequencing, PCR-restriction fragment length polymorphism, real-time PCR, high-resolution melting analysis, loop-mediated isothermal amplification, and single nucleotide polymorphism-PCR have also been used to detect macrolide resistance [36, 70–72]. Liu et al. [71] developed a cycling probe-based method using hybrid cycling probes composed of RNA and DNA, which can rapidly detect *M. pneumoniae* and differentiate resistant strains with good consistency compared to traditional PCR. Commercial kits for detecting MRMP are now available, allowing clinicians to rapidly identify resistant strains, and formulate early treatment plans for pediatric patients with MRMP pneumonia [73–75]. These kits combine PCR with quenching probes (Q probes) to detect target DNA through fluorescence quenching detection of hybridization with fluorescently labeled oligonucleotides, with a turnaround time of approximately two hours. They can not only detect *M. pneumoniae* genes but also detect MRMP mutation genes, such as A2063G and A2064G mutations [75].

### Treatment

#### Antibiotic treatment of MRMP pneumonia

##### Recommendations

Macrolide antibiotics are the first choice for treating MPP. For children aged 8 years and above with MRMP pneumonia, newer tetracycline antibiotics such as doxycycline and minocycline are recommended. For children under 8 years, the use of tetracycline antibiotics requires careful consideration of risks and benefits, and parental informed consent should be obtained. Fluoroquinolone antibiotics are considered as second-line treatment options for suspected or confirmed severe MRMP pneumonia. However, their use in children under 18 years is off-label and requires careful consideration of risks and benefits, along with parental informed consent.

##### Summary of the evidence

Table 1 describes the dosage used for the treatment of MPP [60]. Although a Cochrane systematic review revealed that there was insufficient evidence to support the effectiveness of antimicrobial treatment for pediatric *M. pneumoniae* lower respiratory tract infections, current guidelines still recommend macrolide as the first treatment choice for children with MPP [76].

**Table 1:** Recommended antibiotics for pediatric patients with *Mycoplasma pneumoniae* pneumonia

Medications	Route of administration	Treatment	
		Dosage (mg/kg/d)	Duration (d)
Azithromycin	Oral or IV	10, qd	3–7
Clarithromycin	Oral	10–15, bid	10
Roxithromycin	Oral	5–10, bid	10–14
Erythromycin	Oral or IV	30–45, q8h	10–14
Acetylguitamycin	Oral	25–50, bid	10–14
Levofloxacin			
6 mon-5 y	Oral or IV	16–20, q12h	7–14
5–16 y		8–10, qd	
Adolescents		500, qd	
Moxifloxacin	Oral or IV	10, qd	7–14
Tosufloxacin	Oral	12, bid	7–14
Doxycycline	Oral or IV	4, bid	7–10
Minocycline	Oral or IV	4, bid (first dose, 4 mg/kg)	10

*IV* intravenous, *qd* once daily, *bid* twice daily, *q8h* every 8 hours, *q12h* every 12 hours

Currently, there remains a lack of MRMP guidelines or consensus. Several guidelines recommended tetracyclines and fluoroquinolones as second-line treatment options for *M. pneumoniae* infections. Guidelines from both Japan and Taiwan Province of China indicate [77, 78] that if fever persists or chest imaging continues to progress after 48–72 hours of macrolide treatment, second-line antimicrobial agents such as fluoroquinolones or tetracyclines should be considered. It was also warned that clinicians should weigh the clinical benefits against potential adverse reactions. The 2022 Japanese guideline [79] continues to recommend macrolides as the first-line antimicrobial therapy for childhood *M. pneumoniae* infections, with levofloxacin (available as a pediatric granule in Japan) or minocycline (for children aged 8 years or older) also considered as suitable for treating MPP.

The efficacy of tetracyclines and fluoroquinolones against MRMP has been confirmed. An early clinical study in Japan involving 150 MRMP-infected patients showed that 41%, 48%, 69%, and 87% of patients defervesced within 48 hours of starting antimicrobial treatment in the azithromycin, clarithromycin, levofloxacin, and minocycline groups, respectively [59]. The average duration of fever after antimicrobial treatment was shorter in the levofloxacin and minocycline groups than in the macrolide group. Another study [80] also suggested that levofloxacin and minocycline were more effective than macrolides in treating MRMP pneumonia.

A meta-analysis in 2017 [81], which included eight studies involving a total of 537 MRMP-infected patients, showed that the duration of fever and hospital stay was shorter in the tetracycline (doxycycline or minocycline) group than in the macrolide (azithromycin or clarithromycin) group [weighted mean difference (WMD) = − 1.45, 95% confidence interval (CI) = − 2.55 to − 0.36, *P* = 0.009; WMD = − 3.33, 95% CI = − 4.32 to − 2.35, *P* < 0.001], and the tetracycline group had a significantly higher treatment efficacy than the macrolide group (OR = 8.80, 95% CI = 3.12–24.82). Regarding defervescence, the tetracycline group showed significant improvement compared to the macrolide group (OR = 5.34, 95% CI = 1.81–15.75 at 24 hours; OR = 18.37, 95% CI = 8.87–38.03 at 48 hours; OR = 40.77, 95% CI = 6.15–270.12 at 72 hours). However, there was no difference in fever improvement within 24 hours between the fluoroquinolone group (tosufloxacin) and the macrolide group (OR = 1.11, 95% CI = 0.25–5.00), although the defervescence rate was higher after 48 hours in the fluoroquinolone group (OR = 2.78, 95% CI = 1.41–5.51).

Regarding the safety of fluoroquinolone use, a consensus statement in 2017 pointed out that although there is no unified consensus on whether fluoroquinolones can be used in children, some product labels or guidelines mention that fluoroquinolones can be used to treat children in special circumstances. The use of fluoroquinolones in clinical practice has been increasing annually [82]. A single-center retrospective study from Japan in 2018 included 51,633 children with MPP, and from 2010 to 2014, the usage rate of macrolides decreased from 62.8% to 50.6%, while the usage rate of fluoroquinolones (tosufloxacin) increased from 4.6% to 22.6% (*P* = 0.001) [83].

The safety of newer tetracyclines is relatively reliable. Compared to traditional tetracyclines, doxycycline is less likely to bind to calcium and has a low risk of tooth staining if used for a short duration [84, 85]. A Chinese expert consensus on the use of tetracycline in 2023 suggested that a short course of doxycycline (≤ 21 days) might be considered for children of all ages, after weighing the benefits and risks, when no other antibiotics are available [86].

#### Use of glucocorticoids in MRMP pneumonia

##### Recommendations

Routine systemic glucocorticoids are not recommended for MRMP pneumonia. For severe or critical MRMP pneumonia, systemic glucocorticoid therapy should be considered. The recommended initial dosage of methylprednisolone is 1–2 mg/kg/day. If there is no improvement after 24 hours of initial dosage treatment, it may be increased to 4–6 mg/kg/day. Once the body temperature returns to normal, clinical symptoms improve, and CRP level significantly decreases, glucocorticoids should be gradually tapered off, typically over a course of 3–5 days. For patients with roentgenographic large consolidation, plastic bronchitis, necrotizing pneumonia, or bronchiolitis obliterans with organizing pneumonia, the duration should be extended appropriately. For patients with significant wheezing and allergic predisposition, or asthma during MRMP pneumonia, inhaled glucocorticoids could be used.

##### Summary of the evidence

In 2020, a meta-analysis incorporating 12 randomized controlled trials (RCTs) [87] involving 1130 children with RMPP found that systemic glucocorticoids combined with azithromycin significantly increased the overall effective rate compared to conventional treatment (OR = 6.37, 95% CI = 4.03–10.07, *P* < 0.001). This treatment also effectively shortened fever duration [standardized mean difference (SMD) = 2.29, 95% CI = 2.70 to − 1.88, *P* < 0.001], reduced hospitalization time (SMD = − 2.19, 95% CI = 3.21 to − 1.17, *P* < 0.001), promoted the resolution of pulmonary inflammation (SMD = − 1.89, 95% CI = 2.38 to − 1.40, *P* < 0.001), and showed no significant adverse reactions (OR = 1.18, 95% CI = 0.71–1.98, *P* = 0.53). The glucocorticoids used in these studies were methylprednisolone at a dosage of 2 mg/kg/day or dexamethasone at a dosage of 0.2–0.5 mg/kg/day for a duration of 3–8 days. The results suggested that glucocorticoid therapy for children with MPP could improve clinical efficacy to some extent and is relatively safe. Another meta-analysis published in 2019 including 24 RCTs with a total of 2365 children with RMPP showed similar efficacy with a dosage of methylprednisolone at 1–2 mg/kg/day or dexamethasone at 0.2–0.53 mg/kg/day for a duration of 3–14 days [88].

The meta-analysis published in 2020 compared the effectiveness of high-dose systemic glucocorticoids (methylprednisolone: 10–30 mg/kg/day) and low-dose glucocorticoids (methylprednisolone: 2 mg/kg/day) [89]. This study included 13 RCTs involving 1049 children with severe MPP (SMPP). It is demonstrated that high-dose systemic glucocorticoids had significantly better clinical efficacy than low-dose glucocorticoids (*R* = 1.30, 95% CI = 1.23–1.38, *P* < 0.05). When compared to low-dose methylprednisolone, high-dose methylprednisolone significantly shortened hospitalization and fever duration, improved lung rales, and accelerated the disappearance of lung shadows, demonstrating significantly better clinical efficacy for high-dose systemic glucocorticoids. No significant difference was found in the incidence of adverse events between these two groups.

In a retrospective analysis conducted in 2014, 110 children with RMPP received methylprednisolone treatment at a dosage of 2 mg/kg/day, which resulted in rapid improvement of clinical symptoms and imaging manifestations for most children with RMPP [90]. However, if RMPP patients had persistent fever > 7 days, initial CRP ≥ 110 mg/L, neutrophils > 0.78, lymphocytes ≤ 0.13, LDH ≥ 478 IU/L, serum ferritin ≥ 328 μg/L, and lung computed tomography findings consistent with homogeneous solid shadow above the whole lobe, the 2 mg/kg/day methylprednisolone treatment might be ineffective.

However, not all studies showed the effectiveness of systemic glucocorticoids for MPP, and some risks might exist. A large-scale retrospective study in Japan in 2018, which involved 51,633 children with *M. pneumoniae* infection, showed that among the 12,758 children treated with glucocorticoids, the risk of rehospitalization within 30 days was significantly higher compared to the 38,875 children in the non-glucocorticoid group (1.64% vs. 1.20%; *P* = 0.003) [83].

The recently published Chinese guideline stated that systemic glucocorticoids are mainly used for severe and critical pediatric cases [60]. The recommended dose of methylprednisolone is 2 mg/kg/day, but for some severe cases, adjustments may be made up to 4–6 mg/kg/day, based on clinical manifestations, affected lung lobe number, lung consolidation range and density, CRP and LDH levels, experience, or efficacy. In a few children with severe conditions and overactive immune inflammatory reactions or even cytokine storms, higher doses or even pulse therapy might be needed.

Daily evaluations of efficacy are necessary. If effective, body temperature usually decreases or normalizes after 24 hours of use. If the temperature reduction is not as expected, factors such as inadequate dosage, mixed infection, misdiagnosis, and complications should be considered. Once the body temperature returns to normal, clinical symptoms improve, and CRP levels decrease significantly, the dose can be gradually reduced and eventually stopped, with the total treatment duration generally not exceeding seven days.

Regarding inhaled corticosteroids (ICS), the 2018 Chinese expert consensus pointed out that concurrent use of ICS nebulization therapy alongside antibiotic treatment for *M. pneumoniae* infection could alleviate airway inflammation, promote the restoration of ciliated epithelial cell function, and effectively alleviate airway hyperresponsiveness and non-specific inflammation [91]. For children with MPP who have obvious coughing and wheezing, especially with an allergic or asthmatic background, budesonide suspension could be administered at a dosage of 0.5–1.0 mg per dose, combined with bronchodilator nebulization therapy twice daily for 1–3 weeks.

#### Application of bronchoscopy-guided examination and interventional therapy for the treatment of MRMP pneumonia

##### Recommendations

It is not recommended to routinely use flexible bronchoscopy for the diagnosis and treatment of MRMP pneumonia. However, for severely ill children suspected of having mucus plug obstruction and plastic bronchitis, flexible bronchoscopy should be performed early to reduce the occurrence of complications and sequelae.

##### Summary of the evidence

The 2019 Chinese expert consensus on the interventional diagnosis and treatment of refractory pneumonia in children emphasized the role of respiratory endoscopy in pediatric refractory pneumonia by obtaining specimens for pathogen identification and pathology, as well as treating intrabronchial plastic substances [92]. However, respiratory endoscopy including flexible bronchoscopy is a traumatic operation, and strict control of indications is required. The 2023 Chinese guideline on the management of pediatric MPP stated that routine bronchoscopy examination and treatment are not recommended for mild cases [60]. For severely ill children suspected of having mucous plug obstruction and plastic bronchitis, early bronchoscopic intervention therapy is recommended to reduce the occurrence of complications and sequelae.

In a study conducted in 2023 [93], involving 332 cases of RMPP children, all enrolled subjects underwent flexible bronchoscopy examination and bronchoalveolar lavage, with 270 cases diagnosed with MRMP and 62 cases with MSMP. The results indicated that the MRMP group exhibited more severe bronchoscopic manifestations such as mucosal erosion, ulceration, necrosis, and intrabronchial plastic plugs, causing severe and persistent bronchial obstruction and making it necessary to receive bronchial clearance. Another study in 2022, involving 61 cases of MPP, with 38 cases diagnosed with MRMP and 23 cases with MSMP, also showed similar results [66].

Safety of flexible bronchoscopy is another focus of attention. A study in 2023, involving 990 cases of MPP children, of which 278 (30.9%) cases underwent bronchoscopy examination, showed minimal complications associated with the procedure, with only one case experiencing decreased oxygen saturation (SpO_2_ < 85%), which was quickly resolved after ventilation with a bag valve mask. No serious complications such as pneumothorax, pulmonary hemorrhage, or respiratory failure occurred during the operation [94].

#### Application of intravenous immunoglobulin therapy in the treatment of MRMP pneumonia

##### Recommendations

It is not recommended to routinely use intravenous immunoglobulin (IVIG) in the treatment of MRMP pneumonia. However, IVIG therapy may be considered when severe extrapulmonary complications such as central nervous system damage, skin and mucous membrane lesions, hematological manifestations, or other severe extrapulmonary complications occur. It is suggested to administer IVIG at 1 g/kg/day once daily for 1–2 days.

##### Summary of the evidence

At present, evidence for IVIG treatment in MRMP pneumonia is very limited, with most studies being single-center case reports that do not distinguish between MRMP and MSMP. In a single-center study conducted in China in 2009, 26 cases of central nervous system infections caused by *M. pneumoniae* were included. After treatment with IVIG, azithromycin, and dehydration for intracranial pressure reduction, all patients recovered [95]. Other single-center studies have also shown the effectiveness of IVIG in *M. pneumoniae*-associated hemolytic uremic syndrome [96], as well as *M. pneumoniae*-induced rash and mucositis [97].

The only RCT study was conducted in China in 2017 [94], which included 168 cases of RMPP at a single center. The results suggested that patients treated with azithromycin combined with IVIG had a significantly shorter fever duration compared to those treated with azithromycin alone. The efficacy of azithromycin combined with IVIG or methylprednisolone was similar, but both were significantly better than azithromycin alone. It was concluded that IVIG treatment may be beneficial, especially when glucocorticoids are ineffective or contraindicated [98].

Based on existing evidence, the 2023 Chinese guidelines for the diagnosis and treatment of pediatric MPP recommended IVIG administration for severe extrapulmonary complications such as central nervous system involvement, severe skin and mucous membrane lesions, hematological manifestations, SMPP with concurrent adenovirus infection, or when there is evidence of hyperinflammatory response or severe lung injury. The suggested dose is 1 g/kg/day, administered once daily for 1–2 days.

In conclusion, MRMP pneumonia has become prevalent in the Western Pacific region in recent years, resulting in a greater disease burden. Unresponsiveness to three days of macrolide therapy should be considered indicative for MRMP. Early and rapid identification of gene mutations associated with MRMP is now available by PCR and fluorescent probe techniques in respiratory specimens. Although the resistance rate to macrolide remains high, it is fortunate that *M. pneumoniae* still maintains good in vitro sensitivity to second-line antibiotics such as tetracyclines and quinolones, making them a viable option for patients with initial treatment failure caused by macrolide antibiotics. While macrolides are still the preferred medication for MPP, second-line antibiotics should be cautiously considered for children with MPP from high resistence areas, especially older children. At present, clinical studies on tetracycline and quinolone drugs in children are urgently needed to evaluate the effects of these drugs on the growth and development of children. Additionally, it is necessary to develop an antibiotic rotation treatment strategy for MRMP pneumonia to reduce the prevalence of MRMP strains.

## Data Availability

Data sharing is not applicable to this article as no datasets were generated or analyzed during the current study.
